# USP22 knockdown attenuates *P. gingivalis*-induced EndoMT and CNS inflammation: a link between periodontitis and neuroinflammation

**DOI:** 10.3389/fnins.2026.1790970

**Published:** 2026-03-13

**Authors:** Hui Wang, Mengxian Wang, Jiaming Shen, Hao Zhang, Chengwei Duan

**Affiliations:** 1 Department of Stomatology, Nantong First People’s Hospital, Southeast University Affiliated Nantong First People’s Hospital, Nantong, China; 2 Department of Laboratory Medicine, Shanghai Geriatric Medical Center, Shanghai, China; 3 Deparment of Pharmacy, Huai’an Maternal and Child Health Hospital, Huai’an, China; 4 Intensive Care Unit, Nantong Hospital of Traditional Chinese Medicine, Nantong Hospital Affiliated to Nanjing University of Chinese Medicine, Nantong, China; 5 Medical Research Center, Nantong First People’s Hospital, Southeast University Affiliated Nantong First People’s Hospital, Nantong, China

**Keywords:** endothelial-mesenchymal transition, inflammation, lipopolysaccharide, *Porphyromonas gingivalis*, USP22

## Abstract

**Objective:**

Chronic periodontitis is a significant risk factor for systemic disorders. However, the precise mechanism through which the “oral-brain axis” mediates its impact on the central nervous system remains unclear. This study aimed to investigate whether the periodontal pathogen *Porphyromonas gingivalis* (*P. gingivalis*) induces endothelial-mesenchymal transition (EndoMT), thereby disrupting the blood–brain barrier (BBB) and triggering neuroinflammation, and to elucidate the regulatory role of the deubiquitinating enzyme USP22 in this process.

**Methods:**

A chronic oral infection model of *P. gingivalis* was established in mice, which was confirmed by assessing inflammatory factor levels in periodontal tissues. Endothelial tight junction proteins (ZO-1 and Claudin-5), a mesenchymal marker (*α*-SMA), and inflammatory mediators (iNOS) were detected by Western blotting. IL-1β and IL-6 mRNA levels were detected by quantitative real-time PCR. Human brain microvascular endothelial cells (HBMECs) were stimulated with *P. gingivalis*-derived lipopolysaccharide (LPS) to mimic inflammation *in vitro*. Endothelial-specific knockdown of USP22 in the hippocampus was achieved via adeno-associated virus (AAV) delivery to validate its role *in vivo*. STRING bioinformatics tool to investigate its potential downstream pathways.

**Results:**

Oral infection with *P. gingivalis* successfully induced periodontitis in mice and led to EndoMT in the hippocampus, characterized by downregulation of ZO-1 and Claudin-5 and upregulation of *α*-SMA, along with inflammatory activation evidenced by elevated levels of iNOS, IL-1β mRNA, and IL-6 mRNA. USP22 expression was significantly upregulated both in hippocampal tissues of *P. gingivalis*-infected mice and in LPS-exposed HBMECs. Knockdown of USP22 attenuated LPS-induced EndoMT and suppressed the expression of inflammatory markers in HBMECs. Similarly, endothelial-specific knockdown of USP22 mitigated *P. gingivalis*-induced EndoMT and hippocampal inflammation *in vivo*.

**Conclusion:**

These findings demonstrate that *P. gingivalis* promotes EndoMT and neuroinflammation in the hippocampus. Endothelial-specific inhibition of USP22 alleviates BBB dysfunction and central inflammatory responses, highlighting USP22 as a potential therapeutic target for neuroinflammatory disorders associated with chronic periodontitis.

## 1 Introduction

Chronic periodontitis is a highly prevalent polymicrobial inflammatory disease and no longer considered a condition confined to the oral cavity. Robust epidemiological evidence has established it as a significant risk factor for systemic disorders, including Alzheimer’s disease (AD) and related dementias (Ilievski et al., 2018; Wang et al., 2019). The persistent low-grade inflammation and recurrent bacteremia associated with periodontitis are postulated to facilitate the translocation of periodontal pathogens and their virulence factors to distant organs, potentially triggering or exacerbating neuroinflammatory processes (Bendek et al., 2021; Polak et al., 2018). Among these pathogens, *Porphyromonas gingivalis* (*P. gingivalis*), a keystone periodontal bacterium, is strongly implicated in the pathogenesis of chronic periodontitis and has been a major focus in studying the oral-brain axis. Recent studies have revealed that microglial cathepsin B is increasingly induced by lipopolysaccharide (LPS) form *P. gingivalis*. Gingipains produced by *P. gingivalis* play critical roles in neuroinflammation mediated by microglia and cognitive decline in mice (Nakanishi et al., 2020). Additionally, oral infection with *P. gingivalis* has been shown to induce aberrant astrocyte activation and neuroinflammation, as well as neuronal death (Zhang et al., 2018; Liu et al., 2024). These are also the pathogenic mechanisms underlying central nervous system disorders such as Alzheimer’s disease and Parkinson’s disease (Zhang et al., 2025; Qiu et al., 2024; Yu et al., 2025; Zheng et al., 2021). These findings collectively indicate that *P. gingivalis*-mediated chronic periodontitis is capable of triggering central nervous system inflammation and functional impairment. However, the precise underlying mechanisms remain incompletely understood.

Virulent components released by *P. gingivalis*, enriched in outer membrane vesicles (OMVs), can traverse the blood–brain barrier (BBB), thereby establishing a conduit for cellular and molecular communication between the peripheral system and the central nervous system (Zhang et al., 2020; Wu et al., 2025). Although evidence indicates that intravenous injection of *P. gingivalis* in mice can significantly compromise BBB integrity, the specific pathological changes in endothelial cell phenotypes remain uncharacterized (Lei et al., 2023). Existing literature shows that cerebrovascular endothelial cells lose their endothelial characteristics and undergo endothelial-mesenchymal transition (EndoMT) in various inflammatory conditions, which increases BBB permeability and exacerbates disruption of brain homeostasis (Yang et al., 2018; He et al., 2022). Our previous study also demonstrated that repeated low-dose intraperitoneal injection of LPS can induce EndoMT in mouse brain endothelial cells (Huang et al., 2024). Whether *P. gingivalis* is capable of inducing EndMT in brain endothelial cells warrants further investigation.

The ubiquitin-specific peptidase 22 (USP22), a core component of the SAGA transcriptional co-activating complex, is a key regulator of inflammatory gene expression and cellular responses, although its functional role in different diseases may vary significantly, exhibiting either pro-inflammatory or anti-inflammatory effects (Di et al., 2023; Peng et al., 2025). Preliminary evidence suggests that USP22 is involved in LPS-induced glial cell activation and inflammatory responses in mice (Lu et al., 2025). However, its specific role in *P. gingivalis*-induced endothelial-mesenchymal transition has not been elucidated. To better model the pathological process of periodontitis-associated neurovascular dysfunction, we established a chronic oral infection model in mice and this study identifies a critical pathway that regulates *P. gingivalis*-induced BBB dysfunction. In this study, *P. gingivalis* infection upregulated USP22 expression in hippocampal tissue and promoted endothelial-mesenchymal transition. Inhibition of EndoMT effectively alleviated central inflammatory responses in the *P. gingivalis* infection model. These findings suggest a potential therapeutic strategy for central inflammation-related disorders associated with chronic periodontitis.

## 2 Materials and methods

### 2.1 Animals

C57BL/6 J male mice, aged 6–8 weeks, were procured from the Experimental Animal Center of Nantong University. All animals were maintained under specific pathogen-free conditions with strict regulation of ambient temperature and humidity. All experimental procedures were reviewed and approved by the Animal Ethics Committee of Nantong University, and were conducted in full compliance with institutional guidelines for animal welfare.

### 2.2 Bacterial culture

According to a previous study (Dai et al., 2025), the periodontal pathogen *P. gingivalis* (strain W83) was initially cultured on enriched sterile blood agar plates, composed of 3.7% brain heart infusion agar, 2% agarose, 0.05% cysteine, 0.0005% hemin, 0.0001% menadione, and 5–7% defibrinated sheep blood. Incubation was carried out under anaerobic conditions using an AnaeroPack system for 4–5 days. Subsequently, a single colony was inoculated into BHI broth, which contained 3.7% BHI, 0.0005% hemin, 0.0001% menadione, and 0.05% cysteine, followed by an additional 48-h culture period.

### 2.3 Oral infection model

To induce experimental periodontitis, 6–0 silk sutures saturated with *P. gingivalis* were ligated around the maxillary second molars. This procedure was followed by daily oral administration of 100 μL *P. gingivalis* suspension (1 × 10^9^ CFU/mL) for 24 consecutive days. Successful modeling was confirmed through measuring the inflammatory level of periodontal complex tissues.

### 2.4 Adeno-associated virus (AAV) microinjection

Prior to establishing the oral infection model, microinjection of control or USP22-shRNA AAV (CD144 promoter, 1.5 × 10^12^ vg/μL; Sangon Biotech) was performed to target the mouse hippocampus bilaterally. Each hemisphere received 0.8 μL of the viral suspension. Stereotaxic surgery was conducted for precise placement, with the injection site determined by the following coordinates from bregma: anteroposterior, −2.06 mm; mediolateral, ±1.5 mm; dorsoventral, −2.0 mm. The USP22 shRNA was constructed based on the sequence of 5’-CCAGCAUCAAAGAUGUACUTT −3′.

### 2.5 Cell culture

Human brain microvascular endothelial cells (HBMECs) were obtained from YingBioTech (Shanghai, China). HBMECs were cultured in Roswell Park Memorial Institute (RPMI) 1,640 medium (A1049101, Gibco) supplemented with 10% fetal bovine serum (FBS, Z7186FBS, Zeta Life) and 1% penicillin–streptomycin (C125C5, NCM Biotech) at 37 °C in a 5% CO_2_ environment. HBMECs were exposed to 100 ng/mL LPS from *P. gingivalis* (tlrl-pglps, InvivoGen) for 24 h.

### 2.6 Small interfering RNA (siRNA) transfection

The siRNA targeting USP22 were commercially synthesized by GenPharma (Shanghai, China). HBMECs were treated with USP22 siRNA and control siRNA complexed with the GP-transfect-Mate transfection reagent (G04008, GenePharma) for 48 h. The siRNA sequence used to silence USP22 expression was 5′- GCAAGGCCAAGTCCTGTATCT-3′.

### 2.7 Immunofluorescence staining

Mouse hippocampal tissue sections were cut at a thickness of 8 μm and subsequently permeabilized by incubation in 0.3% Triton X-100 for 15 min, followed by blocking with goat serum (ZLI-9056, ZSGB-BIO) at room temperature for 1 h. The sections were then incubated overnight at 4 °C with CD31 antibody (1:200; ab24590, abcam). The following day, the sections were incubated for 1 h with Alexa Fluor 594-conjugated IgG (1:500; A-11005, Invitrogen). The sections were coverslipped using ProLong Gold Antifade Reagent containing DAPI (0100–20, SouthernBiotech) for nuclear visualization.

### 2.8 Real-time polymerase chain reaction (RT-PCR)

Total RNAs were extracted from cell and tissue samples by VeZol Reagent (R411-01, Vazyme). Subsequently, RNAs were subjected to reverse transcription and RT-PCR using RT Super Mix (R423-01, Vazyme) and SYBR qPCR Master Mix (Q712-02, Vazyme). The primer sequences were provided in Table 1.

**Table 1:** Sequences of primers in this study.

Primer	Sequence (5′ > 3′)
Mouse IL-1β forward	GAAATGCCACCTTTTGACAGTG
Mouse IL-1β reverse	TGGATGCTCTCATCAGGACAG
Mouse IL-6 forward	TAGTCCTTCCTACCCCAATTTCC
Mouse IL-6 reverse	TTGGTCCTTAGCCACTCCTTC
Mouse GAPDH forward	AGGTCGGTGTGAACGGATTTG
Mouse GAPDH reverse	GGGGTCGTTGATGGCAACA
Human IL-1β forward	ATGATGGCTTATTACAGTGGCAA
Human IL-1β reverse	GTCGGAGATTCGTAGCTGGA
Human IL-6 forward	ACTCACCTCTTCAGAACGAATTG
Human IL-6 reverse	CCATCTTTGGAAGGTTCAGGTTG
Human GAPDH forward	ACAACTTTGGTATCGTGGAAGG
Human GAPDH reverse	GCCATCACGCCACAGTTTC

### 2.9 Western blotting

Total protein was extracted with RIPA lysis buffer (P0013B, Beyotime) and quantified using the bicinchoninic acid assay. Equal amounts of protein were separated by sodium dodecyl sulfate-polyacrylamide gel electrophoresis and transferred to polyvinylidene fluoride membranes (ISEQ00010, Millipore). After blocking with 5% non-fat milk, membranes were incubated overnight at 4 °C with primary antibodies, followed by 1 h incubation with corresponding secondary antibodies. Protein bands were visualized using enhanced chemiluminescence and quantified with ImageJ software. All primary antibodies were purchased from Proteintech. Their dilution ratios and product catalog numbers were provided below. iNOS (1:1000, 18,985-1-AP), ZO-1 (1:1000, 21,773-1-AP), Claudin 5 (1:1000, 29,767-1-AP), *α*-SMA (1:1000, 14,395-1-AP), and GAPDH (1:1000, 60,004-1-Ig).

### 2.10 Statistical analysis

Statistical analysis was performed using GraphPad Prism 6.0. For comparisons between two groups, Student’s t-test was applied, whereas differences among multiple groups were assessed by one-way analysis of variance (ANOVA). A *p*-value of less than 0.05 was considered statistically significant. All experiments were repeated at least three times, and data are expressed as mean ± standard deviation (SD).

## 3 Results

### 3.1 Oral infection with *P. gingivalis* induced periodontal tissue inflammation

**Figure 1:**
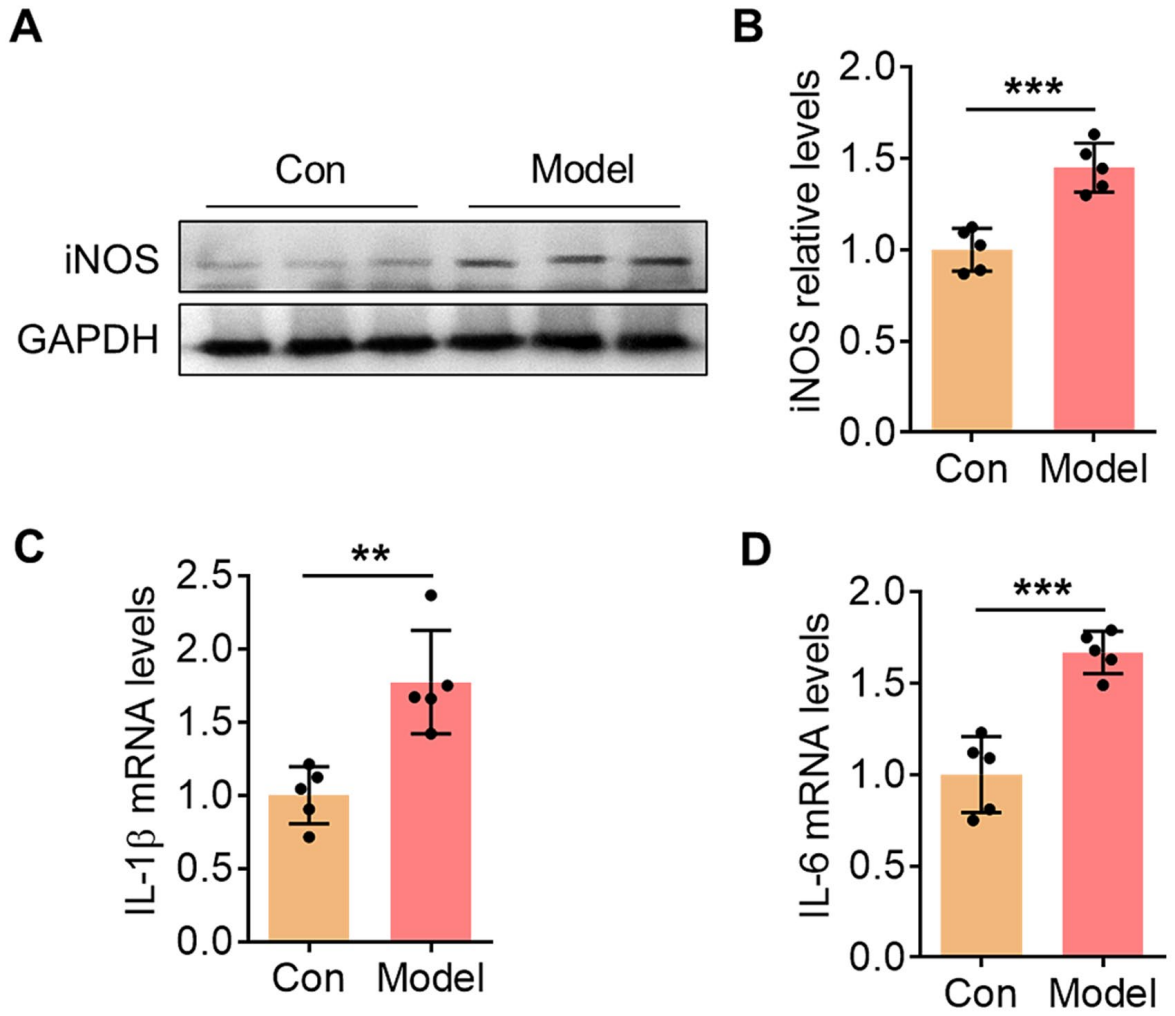
Oral infection with *P. gingivalis* induced periodontal tissue inflammation. **(A)** The representative blot showed the changes in iNOS expression within the periodontal tissues of mice in the control group and the *P. gingivalis*-infected group. **(B)** Bar graph with scatter points showed the quantification of iNOS in **(A)**. **(C)** Bar graph with scatter points showed the quantification of IL-1β mRNA in the periodontal tissues. **(D)** Bar graph with scatter points showed the quantification of IL-6 mRNA in the periodontal tissues. ^**^p < 0.01 and ^***^p < 0.001.

To assess the success of establishing the mouse model, we collected periodontal tissues from the mice and examined the expression of inflammation-related markers. In the periodontal tissues of the model group, the expression of the inflammatory molecule iNOS was significantly upregulated (Figures 1A,B). Additionally, the mRNA levels of inflammatory cytokines IL-1β and IL-6 were also markedly elevated (Figures 1C,D). These results indicate the preliminary establishment of a mouse periodontitis model.

### 3.2 Oral infection with *P. gingivalis* induced EndoMT and inflammation in hippocampus

**Figure 2:**
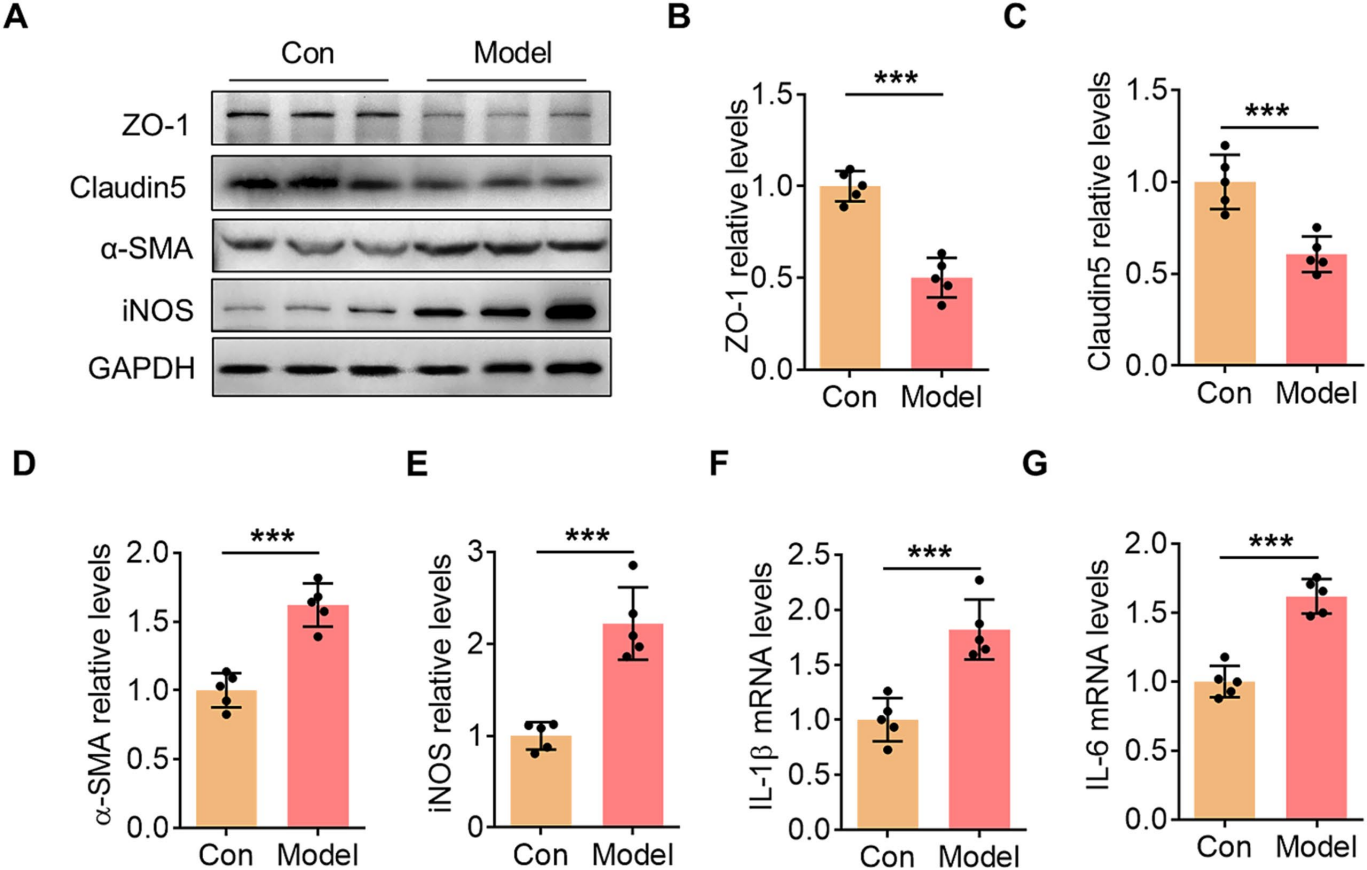
Oral infection with *P. gingivalis* induced EndoMT and inflammation in hippocampus. **(A)** The representative blots showed the changes in ZO-1, Claudin5, *α*-SMA, and iNOS expression within the hippocampus in the control group and the *P. gingivalis*-infected mice. **(B)** Bar graph with scatter points showed the quantification of ZO-1 in **(A)**. **(C)** Bar graph with scatter points showed the quantification of Claudin5 in **(A)**. **(D)** Bar graph with scatter points showed the quantification of α-SMA in **(A)**. **(E)** Bar graph with scatter points showed the quantification of iNOS in **(A)**. **(F)** Bar graph with scatter points showed the quantification of IL-1β mRNA in the hippocampus. **(G)** Bar graph with scatter points showed the quantification of IL-6 mRNA in the hippocampus. ^***^p < 0.001.

We collected samples from the hippocampal region of mice to further examine changes in endothelial cell-related functional proteins and inflammatory levels. In the model group, the expression of endothelial tight junction proteins ZO-1 and Claudin-5 was significantly reduced (Figures 2A–C). In contrast, the expression of the mesenchymal cell marker *α*-SMA increased sharply (Figures 2A,D). Additionally, oral infection with *P. gingivalis* promoted an increase in iNOS protein levels in hippocampal tissue (Figures 2A,E), consistent with the observed upregulation of IL-1β and IL-6 mRNA (Figures 2F,G). These findings indicate that oral infection with *P. gingivalis* induces EndoMT and activates hippocampal inflammation.

### 3.3 Knockdown of USP22 inhibited EndoMT and inflammation in HBMECs

**Figure 3:**
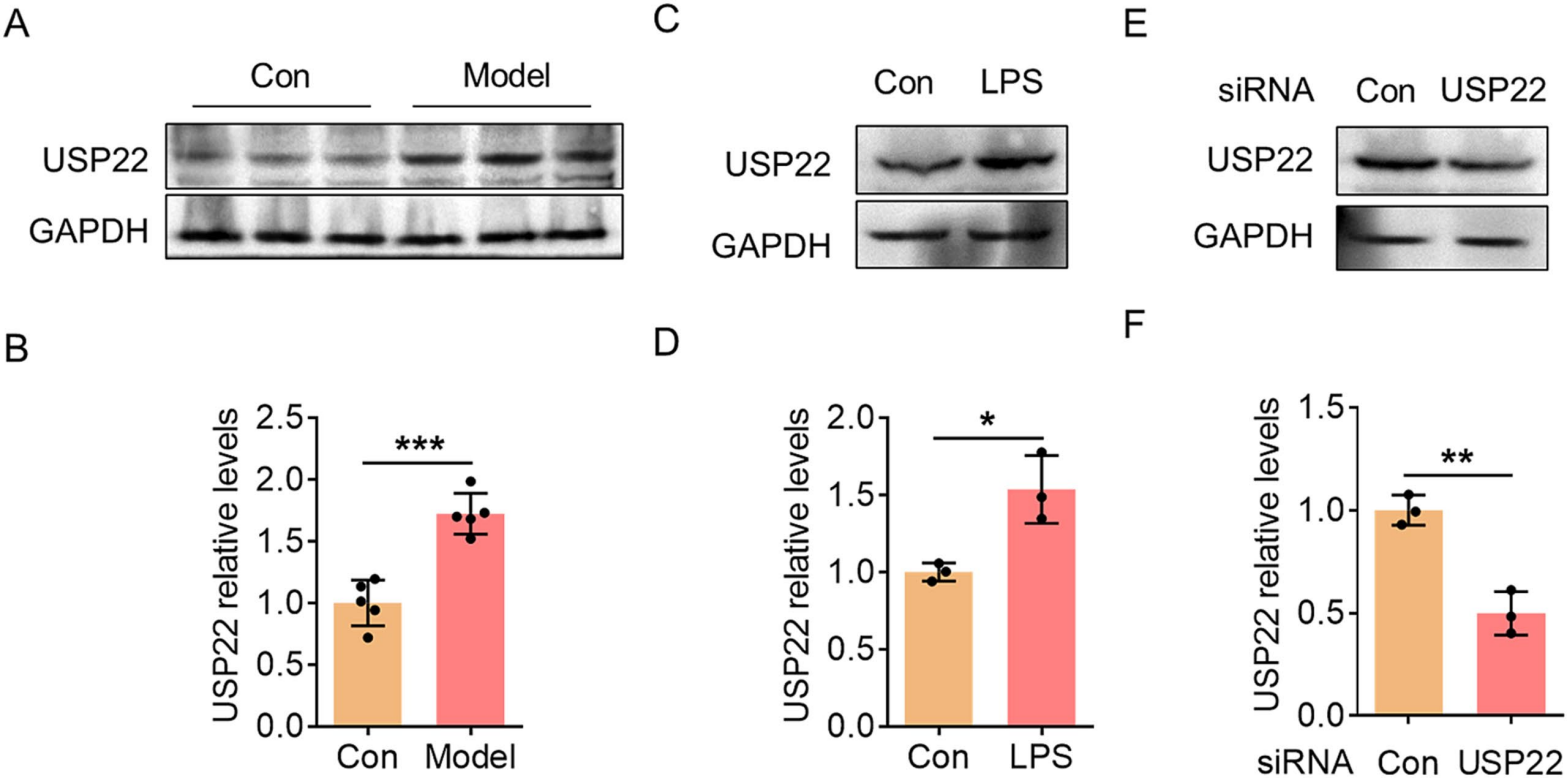
USP22 was upregulated in hippocampal tissues of *P. gingivalis*-infected mice and in LPS-exposed HBMECs. **(A)** The representative blot showed the changes in USP22 expression within the hippocampus in the control group and the *P. gingivalis*-infected mice. **(B)** Bar graph with scatter points showed the quantification of USP22 in **(A)**. **(C)** The representative blot showed the change in USP22 expression in the control and LPS-exposed HBMECs. **(D)** Bar graph with scatter points showed the quantification of USP22 in **(C)**. **(E)** The representative blot showed the change in USP22 expression in the control and USP22 HBMECs. **(F)** Bar graph with scatter points showed the quantification of USP22 in **(E)**. ^*^p < 0.05, ^**^p < 0.01 and ^***^p < 0.001.

**Figure 4:**
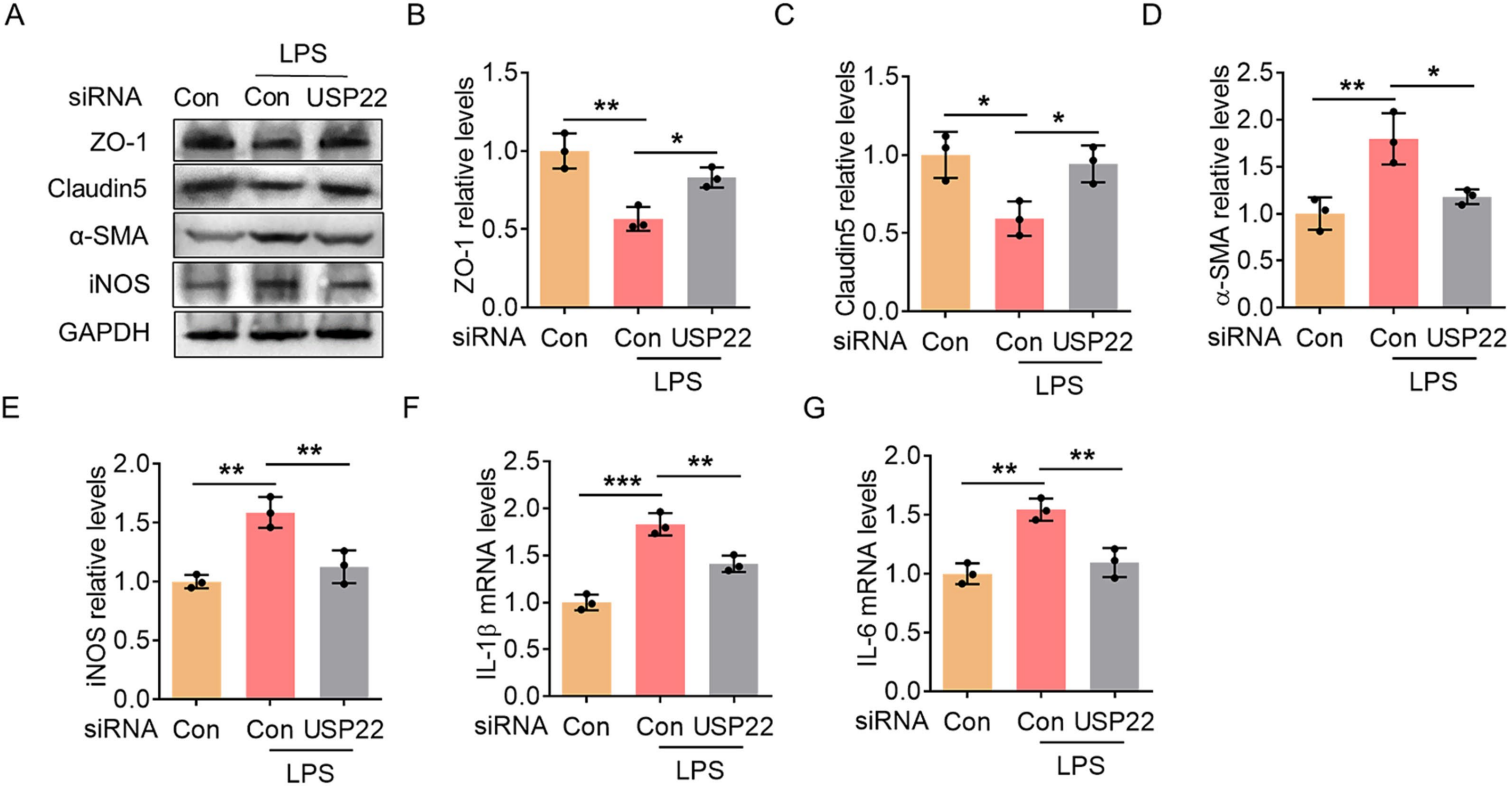
Knockdown of USP22 inhibited EndoMT and inflammation in HBMECs. **(A)** The representative blots showed the changes in ZO-1, Claudin5, α-SMA, and iNOS expression in the control and LPS-exposed HBMECs with/without USP22 siRNA transfection. **(B)** Bar graph with scatter points showed the quantification of ZO-1 in **(A)**. **(C)** Bar graph with scatter points showed the quantification of Claudin5 in **(A)**. **(D)** Bar graph with scatter points showed the quantification of α-SMA in **(A)**. **(E)** Bar graph with scatter points showed the quantification of iNOS in **(A)**. **(F,G)** Bar graphs with scatter points showed the quantification of IL-1β mRNA **(F)** and IL-6 mRNA **(G)** in the control and LPS-exposed HBMECs with/without USP22 siRNA transfection. ^*^p < 0.05, ^**^p < 0.01, and ^***^p < 0.001.

Subsequently, we observed a significant upregulation of USP22 in hippocampal tissues (Figures 3A,B). To simulate the inflammatory condition in endothelial cells *in vitro*, we treated HBMECs with LPS and found that USP22 expression consistently increased (Figures 3C,D). To further investigate this, we designed siRNA targeting USP22 to inhibit its endogenous expression (Figures 3E,F). Experimental results demonstrated that in LPS-treated HBMECs, the expression of ZO-1 and Claudin-5 was markedly reduced, while knockdown of USP22 suppressed this pathological decrease (Figures 4A–C). Conversely, LPS treatment promoted the expression of *α*-SMA, which was reversed by USP22 knockdown (Figures 4A,D). Furthermore, we examined changes in inflammatory markers. LPS treatment elevated the protein expression of iNOS as well as the mRNA levels of IL-1β and IL-6, whereas knockdown of USP22 inhibited the progression of inflammation (Figures 4E–G). These findings indicate that *in vitro* knockdown of USP22 can suppress EndoMT and inflammatory activation.

### 3.4 Endothelial-specific knockdown of USP22 inhibited EndoMT and hippocampal inflammation

**Figure 5:**
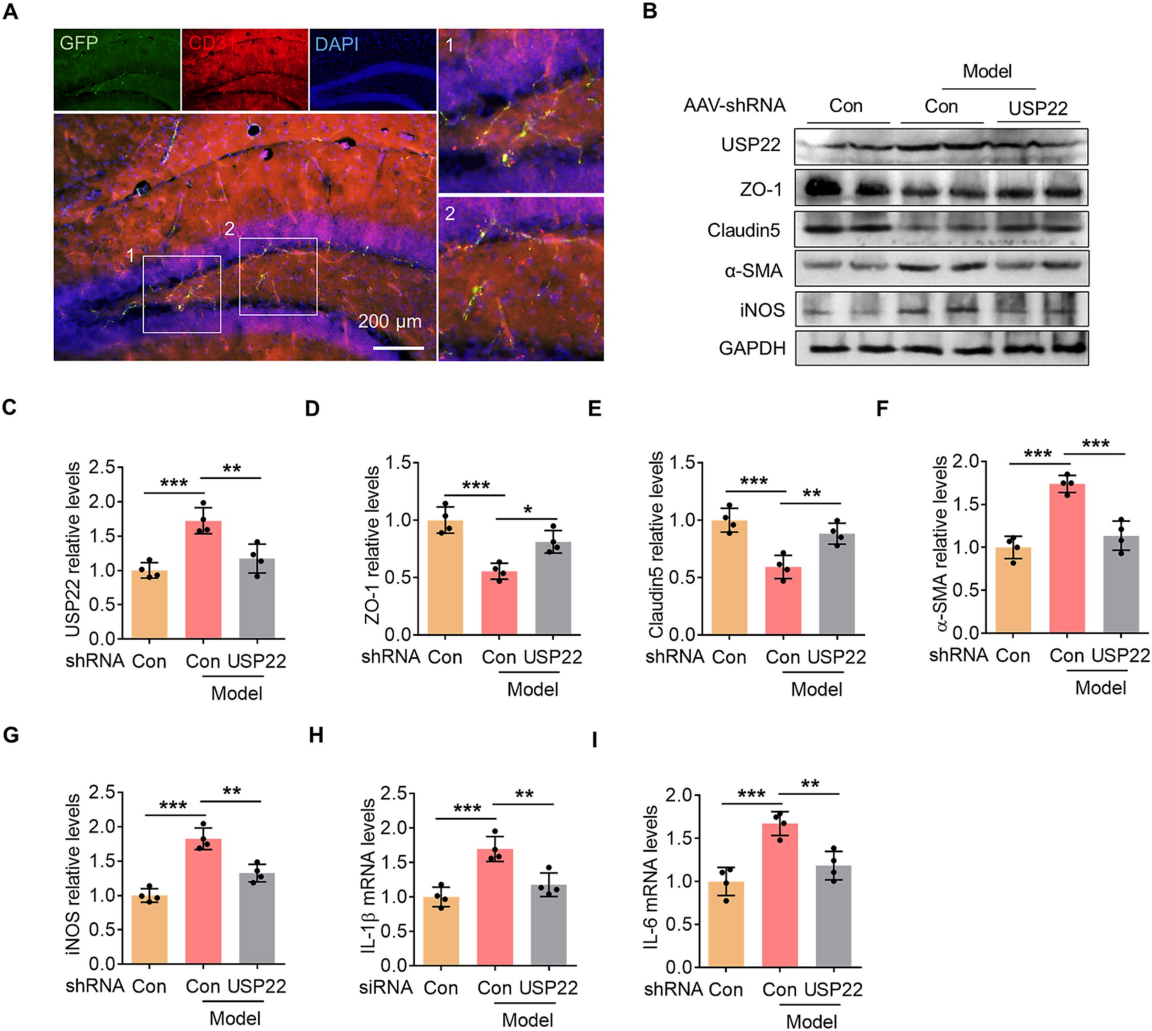
Endothelial-specific knockdown of USP22 inhibited EndoMT and hippocampal inflammation. **(A)** Representative images showed the colocalization of GFP signal (green) and CD31 immunofluorescence (red) in mouse hippocampus. Scale bar: 200 μm. **(B)** The representative blots showed the changes in ZO-1, Claudin5, α-SMA, and iNOS expression within the hippocampus in the control group and *P. gingivalis*-infected mice injected with USP22-shRNA AAV. **(C)** Bar graph with scatter points showed the quantification of USP22 in **(A)**. **(D)** Bar graph with scatter points showed the quantification of ZO-1 in **(A)**. **(E)** Bar graph with scatter points showed the quantification of Claudin5 in **(A)**. **(F)** Bar graph with scatter points showed the quantification of α-SMA in **(A)**. **(G)** Bar graph with scatter points showed the quantification of iNOS in **(A)**. **(H,I)** Bar graphs with scatter points showed the quantification of IL-1β mRNA **(H)** and IL-6 mRNA **(I)** in the control group and *P. gingivalis*-infected mice injected with USP22-shRNA AAV. ^*^p < 0.05, ^**^p < 0.01, and ^***^p < 0.001.

To further confirm the role of endothelial USP22 in *P. gingivalis*-induced hippocampal inflammation, we employed an AAV vector under the control of the CD144 promoter, delivered via hippocampal stereotactic injection, to specifically target endothelial cells. Hippocampal tissue sections were collected and subjected to immunofluorescence staining to assess colocalization between the endothelial marker CD31 and the AAV-carried GFP (Figure 5A). The results showed extensive overlap between GFP signals and CD31 immunoreactivity within the hippocampus, indicating efficient and selective AAV-mediated transduction of endothelial cells. Subsequent analysis revealed changes in endothelial functional proteins and inflammatory markers. In mice orally infected with *P. gingivalis*, hippocampal expression of the tight junction proteins ZO-1 and Claudin-5 was significantly downregulated. This pathological reduction was mitigated by endothelial-specific knockdown of USP22 (Figures 5B-E). Conversely, *P. gingivalis* infection upregulated *α*-SMA expression, which was also reversed by endothelial USP22 knockdown (Figures 5B,F). Furthermore, analysis of inflammatory markers revealed that oral *P. gingivalis* infection elevated iNOS protein levels as well as IL-1β and IL-6 mRNA expression; these increases were suppressed by USP22 knockdown (Figures 5G-I). Taken together, these results demonstrate that endothelial-specific knockdown of USP22 protects endothelial cells from the detrimental effects of oral *P. gingivalis* infection and alleviates associated hippocampal inflammation.

### 3.5 Analysis of the downstream mechanism of USP22

**Figure 6:**
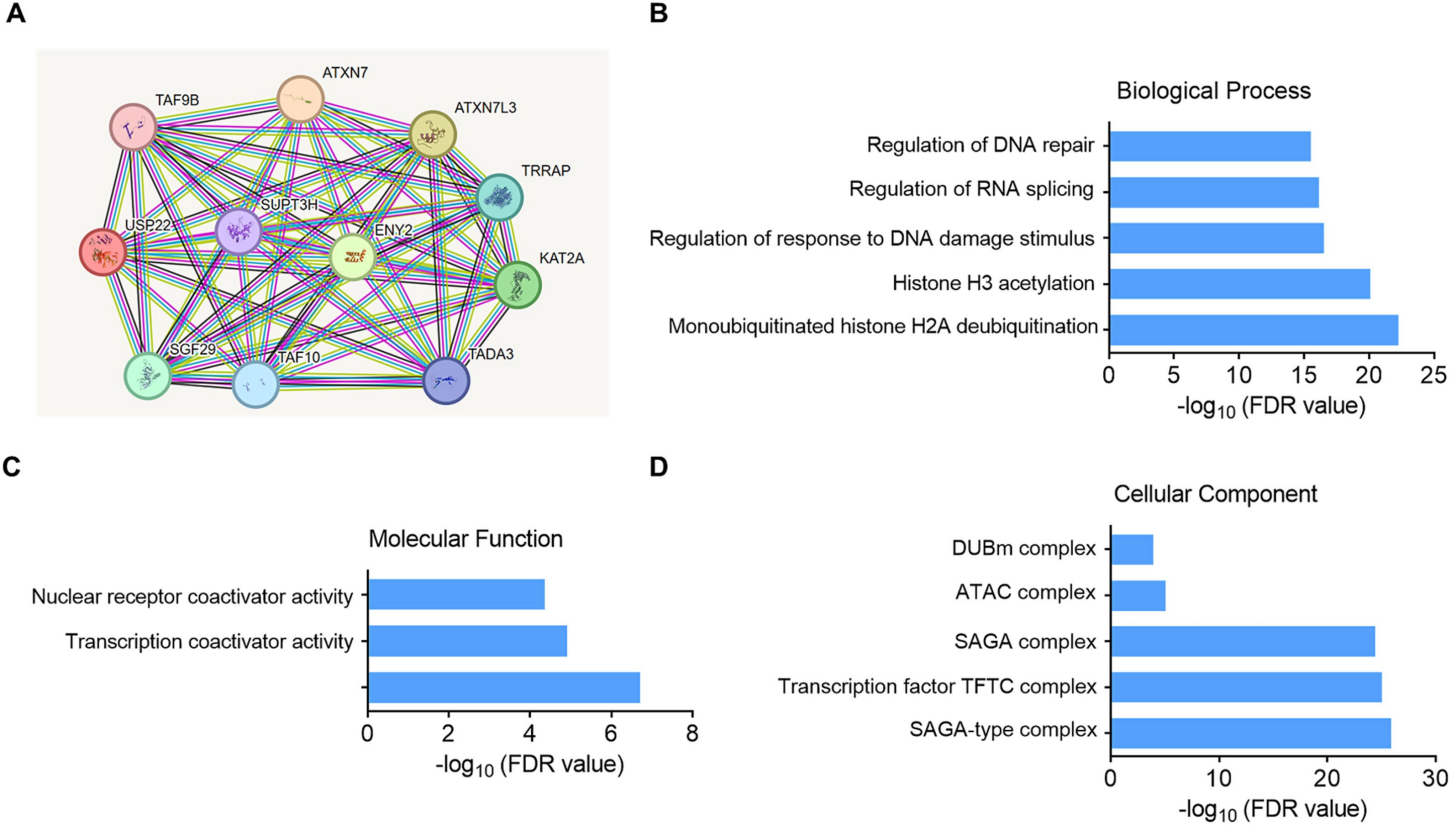
Analysis of the downstream mechanism of USP22. **(A)** Prediction of USP22 downstream-related proteins. **(B)** GO enrichment analysis of biological functions among USP22-associated proteins. **(C)** GO enrichment analysis of molecular function among USP22-associated proteins. **(D)** GO enrichment analysis of cellular component among USP22-associated proteins.

To further analyze the downstream mechanisms by which USP22 regulates EndoMT, we employed the STRING bioinformatics tool to investigate its potential downstream pathways. The analysis identified 10 key proteins associated with USP22. Subsequently, GO enrichment analysis was conducted to elucidate the relevant biological processes, molecular functions, and cellular components. The biological process analysis revealed that the downstream effects of USP22 may be linked to DNA damage repair, histone-related processes, and RNA splicing. Regarding molecular function, the results indicated that USP22 downstream activities may involve nuclear receptor coactivator activity, transcription coactivator activity, and transcription coregulator activity. In terms of cellular components, the analysis suggested that USP22 may function within complexes such as the SAGA-type complex, transcription factor TFTC complex, SAGA complex, ATAC complex, and DUBm complex (see Figure 6).

## 4 Discussion

Chronic periodontitis, a common oral inflammatory disease, is increasingly recognized as a contributing factor to systemic disorders, including neurodegenerative conditions. In this study, we demonstrated that chronic oral infection with *P. gingivalis* induced EndoMT in the hippocampal vasculature, accompanied by significant neuroinflammation. Mechanistically, we identify the deubiquitinating enzyme USP22 as a critical mediator of this process. Endothelial-specific knockdown of USP22 alleviates BBB dysfunction and reduces inflammatory responses both *in vitro* and *in vivo*, highlighting its potential as a therapeutic target in preventing periodontitis-associated neuroinjury.

Previous studies have extensively demonstrated the significant role of USP22 in the progression of various cancers (Shan et al., 2024; Huang et al., 2024). However, a considerable body of literature has also revealed its important function in inflammation-related mechanisms. In aluminum-induced peritonitis and LPS-induced systemic inflammation, USP22 promotes the degradation of NLRP3 and suppresses the activation of the NLRP3 inflammasome (Di et al., 2023). *In vitro* experiments have shown that silencing USP22 enhances inflammatory responses and foam cell formation in macrophages, along with significantly impaired phagocytic capacity (Tang et al., 2025). These findings suggest that USP22 acts as a protective factor that suppresses inflammation. Nevertheless, in renal disease, podocyte-specific deletion of USP22 markedly alleviates angiotensin II-induced podocyte injury and inflammatory responses (Peng et al., 2025). Furthermore, knockout of USP22 in mice significantly ameliorates inflammation and steatosis in a model of alcoholic fatty liver disease (Yan et al., 2021). These studies instead indicate that USP22 functions as a pro-inflammatory factor. In the present study, we found that inhibiting USP22 expression in mouse cerebrovascular endothelial cells suppressed neuroinflammation and EndoMT in the hippocampus. In vitro cellular experiments further confirmed the pronounced pro-inflammatory role of USP22 in HBMECs. The dual role of USP22 observed in different inflammatory contexts may stem from its cell type–specific substrates and signaling environments. This context-dependent functionality underscores the importance of tissue-specific targeting when considering USP22 as a therapeutic intervention.

Chronic periodontitis induced by *P. gingivalis* infection typically persists throughout life. The long-term presence and progressive development of *P. gingivalis* infection gradually exacerbate the accumulation of inflammatory signals and toxic substances in the body (Chen et al., 2023). Substantial evidence indicates that *P. gingivalis* infection can trigger cognitive dysfunction (Jin et al., 2023; Yu et al., 2021). Given that the hippocampal region is a critical brain area for cognition and memory (Lisman et al., 2017; Shibagaki et al., 2024), we aimed to explore the effects of *P. gingivalis* infection on inflammation in the hippocampus. Our study suggests that chronic periodontitis may serve as a previously underappreciated mechanism linking peripheral inflammation to central nervous system injury by inducing EndoMT in the brain. As a source of persistent low-grade systemic inflammation, periodontitis can disrupt the function and structure of cerebrovascular endothelial cells through its pathogen components and circulating pro-inflammatory factors. This study found that targeting endothelial cells in the hippocampal region to inhibit USP22 expression effectively alleviated EndoMT and improved hippocampal inflammation, which supports this hypothesis. Specifically, we propose that periodontitis-associated inflammatory signals may persistently act on cerebrovascular endothelial cells, resulting in EndoMT and neuroinflammation. Such changes may accelerate or exacerbate the pathological progression of central disorders, including Alzheimer’s disease and vascular cognitive impairment (Olsen, 2021; Owoyele and Malekzadeh, 2022). Therefore, targeting EndoMT could offer a novel adjunct therapeutic strategy for mitigating central nervous system damage mediated by peripheral inflammation, such as periodontitis. Future studies should further validate this axis in *in vitro* and *in vivo* models and explore its clinical translational potential. We acknowledge that the present study is limited to a single *P. gingivalis*-based model and focused on USP22 as a mechanistic node. Future studies using additional periodontitis-associated pathogens, as well as in vivo models of periodontitis-neuroinflammation comorbidity, will be necessary to validate the generalizability of our findings. Moreover, the therapeutic potential of targeting USP22 in this context warrants further investigation using pharmacological inhibitors or conditional knockout model.

## 5 Conclusion

Oral infection with *P. gingivalis* induced EndoMT and inflammation in hippocampus. Endothelial-specific knockdown of USP22 inhibited EndoMT and hippocampal inflammation.

## Data Availability

The original contributions presented in the study are included in the article/supplementary material, further inquiries can be directed to the corresponding authors.
